# Functional and Psychosocial Profile of Older People Living in Nursing Homes: Findings from the European Survey of Health, Ageing and Retirement in Europe (SHARE)

**DOI:** 10.3390/healthcare11192702

**Published:** 2023-10-09

**Authors:** Marco Socci, Mirko Di Rosa, Barbara D’Amen, Maria Gabriella Melchiorre

**Affiliations:** 1 Centre for Socio-Economic Research on Ageing, IRCCS INRCA—National Institute of Health and Science on Ageing, Via Santa Margherita 5, 60124 Ancona, Italy; m.socci@inrca.it (M.S.); g.melchiorre@inrca.it (M.G.M.); 2 Centre for Biostatistics and Applied Geriatric Clinical Epidemiology, IRCCS INRCA—National Institute of Health and Science on Ageing, Via Santa Margherita 5, 60124 Ancona, Italy; 3 Italian National Institute of Statistics—ISTAT, Via Cesare Balbo 39, 00184 Roma, Italy; barbara.damen@istat.it

**Keywords:** older people, nursing homes, residential care facilities, health, functional status, psychosocial profile, social network, quality of life, Europe, SHARE survey

## Abstract

Background: This paper is based on results from the Survey of Health, Ageing and Retirement in Europe (SHARE), exploring many aspects (health, economic situation and welfare) of the European population aged 50+. Differently from many other international studies, SHARE includes persons living in nursing homes or residential care facilities as part of its sample. The aim of this paper is to provide a socio-demographic, functional and psychosocial snapshot of older residents in nursing homes in Europe. Methods: This paper uses data from SHARE Wave 8/2020, carried out in 27 European countries. A quantitative/descriptive approach explores the prevalence of older people aged 65+ living in residential facilities as mapped by the SHARE survey across Europe, with regard to associated dimensions, i.e., socio-demographic, family relationship, perceived health/main diseases, functional and psychological status. Results: These show that older residents live mainly in Central and Northern Europe, are aged 80+, female and widowed. A small social network (SN) size is often reported. Health is perceived, above all, as being fair–poor, and the presence of long-term illness is high, with several chronic health conditions and functional limitations. The reported quality of life (QoL) is low for most respondents, with moderate–low satisfaction with life. Conclusion: The analysis depicts a profile of seniors needing residential care in Europe, and provides useful insights for policymakers, to better sustain this frail population group, and to allow and improve access to high-quality long-term care (LTC) in Europe. Our findings could also be of help to train health professionals, and potentially drive the research towards the exploration of new housing solutions for seniors. This would in turn contribute to the effective implementation of European initiatives to strengthen LTC systems.

## 1. Introduction

In 2022, older people aged 65 and over represented 21.1% of the population living in the European Union (EU), with an increase of 3.1 percentage points compared to 2002, and higher shares (more than 23%) observed in Italy, Portugal and Finland [1]. This trend is line with the population ageing process occurring worldwide, that is particularly pronounced in the EU-27, and that is expected to further increase. Indeed, it has been estimated that in the EU-27, the older population will grow in the next few decades, reaching almost 130 million by 2050, compared to 90.5 million in 2019, with a projected increase particularly pronounced in such a timespan among people aged 75–84 years (which number is estimated to increase by 56.1%) than those of the age group 65–74 years (16.6%) [1]. In this context, a key challenge for governments to face is the growing need for health and long-term care (LTC) arising from an (and even more) older population that puts the sustainability of European health and LTC systems under pressure. The latter have already been heavily hit by the COVID-19 pandemic, when the number of deaths due to this infection among older residents in nursing homes was particularly high, especially during the first waves of the health emergency [2,3].

Older people cannot be considered a homogeneous and vulnerable group, dependent and exclusively necessitating health assistance, and therefore a burden for society, even though ageist attitudes, stereotypes and prejudices [4] state this. However, it cannot be ignored that an important part of the older population has increasing caring needs. In fact, older age is often associated with functional dependence, cognitive impairment, frailty, comorbidities and chronic illnesses [5]. For example, as highlighted by the Organization for Economic Cooperation and Development (OECD) [6], across EU countries, on average, about 37% of older people aged 65+ declared to have at least two chronic diseases in 2017, with women tending to report multiple chronic diseases more frequently than men (on average 41% and 32%, respectively). 

Focusing on hospital settings, data from the RePoSi register (REgistro POliterapie SIMI, Società Italiana di Medicina Interna) [7] highlight in particular both the complexity and the importance of the Cumulative Illness Rating Scale (CIRS) assessment of the comorbidity burden in older people hospitalised in internal medicine and geriatric hospital wards, where seniors with comorbidities are recurrently assisted. CIRS (14 items regarding impairment affecting different organs and systems) emerged as a more effective clinical instrument when compared with the easier count of comorbidities, since it includes the length of hospital stay (LOS) and all-cause mortality as proxy variables in this respect. Corrao et al. [7] indeed found that hospitalised older people are often complex inpatients overall, with a high CIRS and treated with more than five drugs. CIRS was also a key predictor of in-hospital mortality. The same authors, moreover, found [8] a “sex dimorphism” in the characteristics of older people hospitalised in internal medicine and geriatric wards, with CIRS and mortality following hospitalisation higher in men, and cognitive impairment and chronic conditions limiting the activities of daily living higher in women. Further similar studies highlighted the importance of clinically significant disability in the oldest hospitalised old patients as a strong predictor of mortality [9]. 

Apart from the hospitalisation context, it is also worth noting that, on average, 3 out of 10 older persons at the EU-27 level reported to suffer from at least one limitation in the activities of daily living (ADLs, e.g., eating, bathing, toileting, dressing) [10] or instrumental activities of daily living (IADLs, e.g., shopping, cooking, managing finances) [11]. Furthermore, the prevalence of experiencing ADL or IADL limitations grows significantly with older age, as almost half of individuals aged 75+ declare having limitations in such activities among EU countries [6]. Additionally, 73.6% of persons aged 85 years and over reported (some or severe) long-standing limitations in their usual activities due to health problems, a share higher than younger age groups and among females [12].

Older people experiencing some kind of limitations that hamper their capability in carrying out the usual activities in daily life, and especially those without an available/fairly robust formal and/or informal caring support network, are exposed to a high risk of becoming frail [13,14]. The latter is “a state of increased vulnerability to poor resolution of homeostasis following a stress, which increases the risk of adverse outcomes” [15] (p. 1). This includes negative health-related outcomes, especially for older people, as frailty is considered as a geriatric syndrome, being a “consequence of age-related decline in multiple physiological systems” [15] (p. 1). In particular, the findings of a systematic review and meta-analysis [16] highlighted that frailty is commonly widespread across Europe, reaching a pooled prevalence among seniors of about 18% in all settings, even though it varies on the basis of settings (e.g., community, primary care, nursing home or residential care facility, hospital) and the definition adopted for frailty. For example, the prevalence of frailty observed in community-based studies on older people ranged between 2% [17] and 60% [18], with an estimated mean prevalence rate of 12% [16]. Instead, according to another systematic review [19], 50% of people aged 60 years and over residing in nursing homes or residential care facilities were categorised as being frail, even though the analysed studies showed a high heterogeneity concerning the mean estimated prevalence of frailty, which ranged from 19% to 76%. Other authors highlighted that older people living in nursing homes experience a higher development of frailty and a worsening of both quality of life and physical wellbeing compared to community-dwelling seniors [20,21]. Also, as suggested by a recent review and meta-analysis [22], frail older people with functional limitations and difficulty in the activities of daily living have limited contacts with others, thus increasing the risk of their social isolation. Moreover, socially isolated seniors were found significantly more likely to be frail when compared with their counterparts [22].

The literature widely pointed out that older people prefer to age in place at home with the support of family members and/or public services (e.g., home care)—a tendency that in several cases increases with age—while other living solutions (e.g., co-housing/multigenerational buildings, moving to other houses) are less considered, especially concerning the willingness/decision to move to a residential care facility/nursing home [23,24,25,26,27,28,29]. However, ageing may require older people to change their living environment, either by adapting their current homes or by moving to more supportive environments, such as LTC facilities [30]. The latter option may occur among older people because of the reduction in/absence of a family support network, an inadequate provision of public home care services, economic difficulties in accessing the private care market, the growing caring needs due to functional limitations in daily/usual activities associated with health problems, chronic illnesses/cognitive impairments and increasing disability and frailty related to age [31,32]. Moreover, family caregivers (mainly women) provide about 80% of LTC across Europe [33], but their availability is negatively affected by social changes such as the growing participation of women in the labour market, the increase in the retirement age and changes in family structures/shrinking in family sizes [34,35,36]. Also, differing cultural norms and attitudes regarding care for older people in Europe need to be considered. In particular, “family obligations” norms and traditions are stronger in Southern European countries (e.g., Spain and Italy), where support comes mainly from seniors’ own households. Conversely, in Northern Europe (e.g., Scandinavian countries), this aspect is weaker, with support also often coming from outside the family (e.g., LTC formal services) and co-residence with children is less frequent [37].

The “combined effect” of the overall aspects mentioned above, in particular the wide and growing LTC needs of older people due to population ageing, has an impact on LTC public expenditure as a percentage of gross domestic product (GDP) in the EU-27, that increased from 1.54% in 2014 to 1.8% in 2020 and it is expected to grow to 2.9% of GDP in 2070, i.e., equating to an increase in expenditure by more than 70% during this time span [31]. Moreover, the supply of LTC services, e.g., number of beds in LTC residential care facilities, is managed differently according to the characteristics of the European LTC systems that have been classified in a continuum from the services-led model, characterising Nordic countries, to the informal care-led model, typical of Southern countries, with Central countries in an intermediate level and Eastern countries tending to be near to the first side/model [38,39,40,41]. In Europe, a significant share of healthcare expenditure is spent on residential LTC facilities, i.e., 10.2% in 2018 [42]. However, the availability of residential/institutional care to older people varies significantly among EU countries. In fact, the number of LTC beds in nursing homes and residential care facilities is higher in Central/Continental and in Northern European countries and lower in Southern and Eastern EU Member States [31,32,43]. 

It has also been estimated that overall, throughout the EU-27 in 2020, there were around 3.4 million LTC beds in nursing homes and residential care facilities [44], but between 2010 and 2020, in only 7 out of 24 countries for which data were available was there a growth concerning such an indicator [45]. Thus, in the majority of the EU Member States (especially in Nordic countries), for at least 10 years, there have been policies in place aimed at sustaining a process of de-institutionalisation, i.e., a shift from residential care to community/home care [36], and in particular to support ageing in place in the homes of older people, largely motivated by cost-effectiveness. However, there could be the possibility of an increasing demand for residential care in the near future, mainly in the case of a further decreasing propensity and supply of informal carers to provide care to their loved ones with LTC needs [32,43,46]. Also, on average, in the EU-27 in 2020, only 3.6% of older people aged 65+ received institutional care [32], while, between 2010 and 2020, among the 19 EU countries for which data were available, only in five cases was an increase in the recipients aged over 65 (in the percentage of the total population aged 65+) in LTC institutions observed [45]. This highlights a decrease in the coverage of LTC facilities for older people in need of care or confirms a trend of policies sustaining community care and ageing in place for frail older individuals. Such a situation is substantially in line with the trend described above of the overall reduction in available beds in LTC facilities across European countries. Despite the prevailing approach mentioned above of sustaining a shift towards community care, a report published by Eurofound [47] showed an increase in the number of care homes (i.e., nursing and residential care homes) in the large majority of the 20 European countries analysed in its study, from about 10 years ago, especially private care homes compared to public ones. 

Within such a depicted framework, it has to be noted that in several countries, the decision to move to nursing homes/residential care facilities is not considered as a desirable/last option for most older people. This is not only due to the higher costs, but also as it can be a critical and stressful life experience, because it may lead to a loss of independence, autonomy, identity, social contacts and usual habits for seniors having to leave their usual home environment [23,29,48,49]. Previous research has also pointed out that living in nursing homes is related to the low socio-economic status of seniors, e.g., low educational level and low income/economic resources [41]. In addition, the institutional characteristics of LTC systems, i.e., the availability of beds in state-subsidised LTC facilities, regulated by increasing means-testing measures, may play a role in determining a higher prevalence of such older people living in nursing homes [41].

In the literature, few evidence on the socio-demographic profile and characteristics of older people living in LTC facilities (nursing homes and residential care facilities/institutions), or other aspects of seniors living in such kind of arrangements [21,41,50], are available. This is mainly due to the difficulty in carrying out surveys in such “protected” contexts (and especially it was the case during the COVID-19 pandemic) and by retrieving and using comparative and reliable data.

To fill this gap in knowledge, the present study aimed to investigate the functional and psychosocial profile of older people needing LTC living in nursing homes/residential care facilities in Europe, drawing on data available from the Study of Health, Ageing and Retirement in Europe (SHARE), Wave 8/2020 [51,52]. In particular, this paper aims to answer the following research questions:

1. What is the prevalence of persons aged 65+ living in nursing homes, as mapped by the SHARE survey in Europe?
2. What is the socio-demographic, functional and psychosocial snapshot of older residents in nursing homes, as captured by the SHARE survey in Europe?

The results of the analysis can contribute to providing an updated profile of older people needing residential care in Europe, and to offer useful insights for policymakers to better sustain such a frail population group. 

## 2. Materials and Methods 

### 2.1. Data Source: The SHARE Survey

As anticipated in the Introduction, this study is based on results from the SHARE study [53], a cross-national biannual longitudinal survey, with repeated waves over years since 2004. It regularly explores several aspects (health, socio-economic situation, welfare) of the population aged 50 years and over in several European countries, and provides useful insights for policymakers, also with regard to older people [54]. Differently from many other international studies, SHARE includes persons living in nursing homes and residential care facilities, as part of the general population sample [52]. This study uses, in particular, data from SHARE Wave 8/2020 [51,52], with a quantitative approach to measuring the number/prevalence of older people aged 65+ living in residential facilities in Europe captured by such a data source, and analyses associated socio-demographic, family, health, functional/psychological aspects. In Wave 8, 27 European countries (and Israel) were examined: Austria, Belgium, Bulgaria, Croatia, Czech Republic, Cyprus, Denmark, Estonia, Finland, France, Germany, Greece, Hungary, Italy, Latvia, Lithuania, Luxembourg, Malta, the Netherlands, Poland, Portugal, Romania, Slovakia, Slovenia, Spain, Sweden and Switzerland [55]. Regarding Wave 8, more detailed information on available variables and datasets can be found elsewhere online [56].

The SHARE design uses probability sampling, and assures a maximal population coverage [54,57]. It is anyway worthy to clarify that, due to the COVID-19 pandemic that affected Europe in early 2020 (which, according to the WHO, ended as a public health emergency in May 2023) [58], and the related strict control measures/restrictions and lockdown, SHARE face-to-face interviewing at the homes of respondents, i.e., via computer-assisted personal interviewing (CAPI), initiated in October 2019, was suspended in all 28 participating countries in March 2020. Then, conversely, computer-assisted telephone interviews (CATIs) were administered, continuing to collect data but only on some/most important topics of the regular SHARE questionnaire, and focusing especially on the health and living situation of people aged 50+ during the pandemic (SHARE Corona questionnaire, June–August 2020) [52,55]. This circumstance, i.e., moving from in-person to telephone interviews, could be partly responsible for the high number of missing answers in some dimensions of the overall Wave 8. 

### 2.2. Definitions, Measures and Variables Used for the Study

The reference variable is the following: “NursHome (NURSING HOME)” [59,60]: “Does the eligible respondent live in a private household or in a nursing home?” In SHARE, a nursing home “provides all of the following services for its residents: dispensing of medication, available 24-h personal assistance and supervision (not necessarily a nurse), and room & meals” [61] (p. 254). Thus, since this category seems to include any LTC facility for older persons, both definitions “nursing home” and “residential care” can be used. In other words, nursing homes are facilities for older people who need support in the activities of daily living, and where they receive care for short or long periods. Moreover, the OECD [62] states that residential LTC facilities comprise establishments primarily engaged in providing residential LTC that combines nursing, supervisory or other types of care as required by the residents. The topics and further variables used in this study are summarised in Table 1.

Among socio-demographic characteristics, educational level was measured by the number of years spent attending full-time education, in addition to the International Standard Classification of Education (ISCED-2011). This coding includes 8 levels [63]: level 1 = primary education; level 2 = lower secondary education; level 3 = upper secondary education; level 4 = post-secondary non-tertiary education; level 5 = short-cycle tertiary education; level 6 = bachelor’s or equivalent level; level 7 = master’s or equivalent level; level 8 = doctoral or equivalent level. These levels have been further grouped into three main categories: primary education or less (level 1), secondary (levels 2–3) and post-secondary (levels 4–8). These main three categories also include the six levels of the previous ISCED 1997 [64,65]. 

With regard to SN size, “network less” means that respondents reported no persons in their SN (family members, friends, neighbours, acquaintances) with whom they discussed important issues within the last year; “small” means that respondents reported one–two members; “large” means that respondents reported three or more members [66,67]. The inventory SN satisfaction ranges from “0” as completely dissatisfied, to “10” as completely satisfied with the relationships [68,69]. It was further grouped in three main levels: low (0–6), moderate (7–8) and high (9–10) [70]. 

Concerning the functional status, six basic ADL (dressing, walking across a room, bathing, eating, getting in or out of bed and using the toilet) and seven IADL (using a map, preparing a hot meal, shopping for groceries, making telephone calls, taking medications, undertaking work around the house or garden and managing money) were included [70]. Mobility limitations generally means “arm function and fine motor limitations” [70] (p. 2865).

With regard to QoL, the Control, Autonomy, Self-realization and Pleasure (CASP-12) scale/index was used in SHARE. It contains 12 items (and 4 dimensions: control, autonomy, self-realisation and pleasure), which are rated on a four-point Likert-scale (often, sometimes, not often and never) [71]. The total score ranges from 12 to 48, with higher scores corresponding to a better QoL. Cut-offs are usually put at <35 (low) and ≥35 (high) [68,70]. Regarding satisfaction with life, “0” means completely dissatisfied and “10” completely satisfied [68,69]. It was further grouped in three main levels: low (0–6), moderate (7–8) and high (9–10) [70]. Regarding other psychological aspects, responses/feelings/opinions were categorised as Yes/No, often/sometime/rarely/hardly ever or never and do not know.

Missing values are reported (as footnotes in the tables) when n ≥ 150 (apart from Table 2). Also, for each dimension, in the tables, only the higher values (only first, first to second, first to third, depending on the topic, with some exception, i.e., all values) are reported.

### 2.3. Data Analysis 

Our study focused on people aged 65+ years living in nursing homes/residential care facilities. Thus, after filtering respondents belonging to this age group and living in a nursing home, within the whole SHARE (Wave 8/2020) sample of people aged 50+ (n = 57,446) [52], we obtained a sub-sample of 485 seniors. The findings were then differentiated according to several dimensions (cited above, Table 1). The main results are presented in seven tables with regard to the following topics: preliminary aspects of nursing homes (e.g., years in accommodation); socio-demographic characteristics; family; SN; health; functional status, ADL, IADL and use of aids; and quality of/satisfaction with life and other psychological aspects. This allowed for providing a detailed/descriptive profile of seniors needing residential care in Europe via the means of frequency distribution/descriptive analyses. It is worth highlighting that previous studies analysed residents aged 65 years and over in nursing homes by using SHARE pooled data from waves 5/2013, 6/2015 and 7/2017, but only across 12 European countries (Austria, Belgium, Czech Republic, Denmark, France, Germany, Italy, the Netherlands, Slovenia, Spain, Sweden and Switzerland) [41]. They found, on the whole, 1198 individuals (about 400 seniors for each wave) vs. 485 in Wave 8/2020 and 28 countries. Probably, without the impact of the COVID-19 pandemic that posed a serious risk, especially for older people [72], with deaths registered mainly in nursing homes/residential care facilities [73], the share of seniors in nursing homes covered by the SHARE Wave 8/2020 survey could have been greater.

## 3. Results 

### 3.1. Length of Stay and Socio-Demographic Characteristics of Older Residents

Older residents in nursing homes mainly report short periods in this accommodation (61.2% up to six years) (Table 2).

Moreover, about 72% of them live in Central and (mainly) Northern Europe, whereas the n. values registered in the countries in the South and the Mediterranean area were particularly low in Greece (2), Cyprus (2), Italy (3) and Croatia (5), and higher in Spain (27). In total, 73% are aged 80 years and over, 68% are female, 55% are widowed and 83% report a small household size (i.e., one member). Regarding education, primary/secondary levels prevail, and public old-age pension represents the main income source for about 84% of residents. Also, 64% use their pensions to cover the cost of the residential care facility (Table 3).

### 3.2. Family Members

Few older residents report at least one brother and/or one sister still alive (respectively, 22% and 24%). Similarly, 23–30% report one–two children and 22–24% report four–five and two–three grandchildren (Table 4).

### 3.3. Overall Social Network (SN)

A small SN size is mainly reported (one–two members for 50% of respondents), even though the SN satisfaction is high for about half of the sample. Children (45%) are the most cited persons in the network, that is made mainly by women (54%). Daily contacts are reported by 37% of respondents (Table 5).

### 3.4. Health 

Health is perceived, above all, as being fair–poor (37–31%), and a good–fair memory is reported (about 28% for both levels). However, the presence of long-term illness is high (77%), with 41% suffering from three–six chronic health conditions, mainly high blood pressure or hypertension (39%), and half of residents take at least five different drugs in a typical day. Also, 76% wear glasses/contact lenses, and about 50% report troubles with pain (Table 6).

### 3.5. Functional Status, ADL, IADL and Use of Aids

About 57% of older residents are severely limited in activities because of health. Almost 41% present three–six+ ADL limitations, more than half have six and more IADL limitations and 40% have five–eight mobility limitations. Moreover, the use of aids is frequent, mainly Zimmer frame or walker, and incontinence pads (40% both). Also, dizziness/faints/blackouts, fear of falling down and fatigue represent the principal aspects affected by frailty (38–37%) (Table 7).

### 3.6. Quality of/Satisfaction with Life and Other Psychological Aspects 

Regarding QoL, and apart from many missing data (n = 174), the majority of respondents report a low QoL (36%) and a moderate–low satisfaction with life (29–27%). On the whole, older residents sometimes/rarely do the things they want to do, feel full of opportunities and think that the future looks good. Moreover, they rarely/never feel full of energy, and often/sometimes, they consider age as preventing them from doing things. As a possible consequence of such a context, they felt sad/depressed last month, even though they hardly ever or never felt isolated from others and lonely, and some hopes for the future were, however, mentioned. Conversely, suicidal feelings or a wish to be dead were not mentioned by the majority of respondents (Table 8).

## 4. Discussion 

The aim of this study was to provide a socio-demographic, functional and psychosocial profile of older European people aged 65+ living in nursing homes/residential care facilities, as mapped by the SHARE survey, Wave 8/2020. Our results showed that they are mainly aged 80 years and over, female, widowed, living in Central/Northern Europe, with a low educational level and with a small SN size. These seniors also report how health is perceived, above all, to be fair–poor, and several chronic health conditions and functional limitations are reported, in addition to both a low QoL and satisfaction with life in many cases. From the overall analysis, insights for policymakers emerged with regard to the necessity to better support older people needing residential care in Europe, as described in more detail below. 

### 4.1. Length of Stay in Nursing Homes and Socio-Demographic Profile of Older Residents

A first aspect to emerge from the SHARE data is that a great part of older residents in nursing homes are living there at most from one–six years. According to some authors [74], 65% of residents in nursing homes turn to a longer stay (over 90 days from admission), whereas the remaining 35% are discharged to the community/hospital or died (within 90 days from admission). Other authors [75] highlighted that nursing home lengths of stay are less than six months for the majority of residents at the end of life, with decreasing values (shorter median lengths of stay) for seniors with chronic illnesses and functional impairments. This overall context probably also relates to the quite high age of residents (better explained below).

Regarding the country/area of origin, SHARE data indicate that about 62% of older residents live in Central and (mainly) Northern Europe. OECD data for 2020 [45] show that available beds, for 1000 seniors aged 65 years and over in need of LTC, are 18 in Italy, 70 in Sweden and 75 in the Netherlands. Stolz et al. [50] also found that 2.1% of the older population in Greece and 3.5% in Poland, vs. 27.9% in Switzerland and 29.7% in Denmark, accessed a nursing home in the last year of life. Overall, several authors confirmed that the availability of residential care differs greatly across Europe. Indeed, LTC systems differ, with informal care prevailing in Mediterranean/Southern countries, formal care/services/facilities prevailing in Scandinavian/Nordic countries and Central European countries overall put in the middle [41,61]. Stolz et al. [50] further confirmed that different public spending on institutional care across European countries impacts and differentiates access to nursing homes, and that access for seniors to nursing homes could in turn be facilitated in countries with a better/more efficient public LTC system. Generous public funding for residential care prevails in Northern countries (Sweden, Denmark); conversely, scarce publicly funded facilities and related limited available beds prevail in Southern Europe [41]. Other authors [76] found in particular that differences in public spending lead in turn to differences in available care home infrastructure, with higher opportunities for accessing adequate nursing homes mainly in Sweden and the Netherlands. It is also worth highlighting that the previous literature reported how institutional care is a marginal option in Southern and Eastern European countries, especially for older people with no available caring relatives, whereas in North-western Europe, nursing homes represent a “more viable option in case of substantial care need” [50] (p. 8). Further authors, similarly, found that moving to a nursing home represents a last option if there is a caring family [29], and reported in particular that co-residence with children represents an alternative to nursing homes in countries with a low availability of facilities for seniors [61]. Thus, cross-country/cultural variations also impact the access to nursing homes (e.g., familistic culture in Mediterranean countries) [77]. 

Concerning other socio-demographic aspects, the overall “SHARE picture” of older residents in nursing homes puts in evidence the following: high age, female gender, widowhood, small household size (one member) and primary/secondary education. In this respect, interestingly, some authors [21] compared the socio-demographic profile of frail older people living at home or in a nursing home, and found mainly in the latter the oldest people, both genders, widowed, with a low education and those who lived alone. Holup et al. [74] similarly reported that residents accessing a nursing home were mainly female, widowed, with an average age of 84 years and living alone for about 30%. Other authors [78,79] also put in evidence older age and living alone as individual risk factors for admission to residential facilities. Regarding age, the literature highlighted that moving to a nursing home is more likely after the age of 85 years [61], whereas seniors living at home are younger. In particular, entering a nursing home before 75 years of age is almost infrequent, and it can happen at least when older people have some functional limitations and poor informal support, also following poor/lacking contact with their families [80]. Regarding education, Bachmann et al. [81] found that older people with the lowest level had a 22% higher likelihood of entering a nursing home than those with higher education levels and attributed this to the link between low education and a higher risk of not managing chronic health conditions well. Regarding living alone, the same authors [81] found that such seniors have a 55% higher likelihood of accessing a nursing home, than those living with others. Also, the previous literature highlights specifically that the presence of a spouse reduces the probability of entering a residential care facility, independently from the health status [61,78]. The death of a spouse thus represents a crucial/setting-off moment for seniors [82]. Some authors [81] analysed, in particular, the critical discharge from hospital in later life as a crucial moment especially for socially deprived seniors, who probably will access a nursing home due to LTC needs and the specialist support required that family members (even with the help of private care assistants) are unable to guarantee at home. In this regard, further authors reported that older residents discharged from hospital to a nursing home were, above all, old women, widowed and living alone [83]. In a study [74], these seniors were 85% women and 56% widowed after a long stay in hospital. Other authors [84] found living alone as a key predictor of non-home discharge for seniors after a long hospital stay, with possible admission in an LTC facility.

Regarding economic status, our results indicate that seniors in a nursing home have a public old-age pension as the main income source, and that they mainly use their pensions to cover the cost for such an arrangement. Several authors stressed these aspects and found a poor economic context (without a further income source, e.g., annuities) emerging mainly for seniors living in an LTC institution, than those living at home [21]. Especially across European countries, seniors in the lower quarter of financial resources were more likely to access a nursing home [61]. Some evidence from the literature also reports a strong link between living in a nursing home and a limited disposable income of residents [79], and that 45% of seniors admitted in a nursing home relied on their own or family financial resources for payment [74].

### 4.2. Family Members and Overall SN

The presence of family members of older residents in a nursing home does not emerge as broad/extended from the SHARE data. Few respondents indeed report a partner and at least one brother and/or one sister still alive, and mainly one–two children, and two–three grandchildren. Accordingly, some of the literature [80] indicates that seniors living in a nursing home are more frequently without children, brothers and/or sisters still alive. Also, Hrast et al. [82] state that childless people are more likely to be admitted in a residential facility. Regarding children, some of the literature specifies that only the presence of a living-in/nearby daughter decreased the likelihood of moving from their own home to an institution [61]. However, this replacement generally seems linked to the absence of relatives living close to seniors needing help [30].

SHARE findings further highlight a small SN size for these older residents, with half of them reporting one–two members, mainly children and female relatives. Following the previous literature, overall, a low availability of informal help (e.g., mainly spouse and children) represents a key predictor of institutionalisation [61]. Indeed, seniors living in a nursing home more frequently have limited informal support, in particular from/within the household [61,80], and the moving of seniors to an institution is often due to their severe care needs not being met with adequate support [82]. Decisions regarding housing solutions in later life are thus also dependent on an available SN [30]. Other authors [78,79] confirm a scarce social support as an individual risk factor of nursing home admission, whereas conversely, informal care availability prevents accessing a nursing home in countries with a lower public LTC spending in this respect [50]. Family is indeed an effective support that can substitute access to a nursing home when the LTC needs of seniors are low [85]. However, relatives could be present but not able or available to take care of seniors, thus requiring an intervention of formal/institutional support. Roquebert and Tenand [80] in particular put in evidence that among older residents in a nursing home (aged 60 years and over, with functional limitations), 76%, however, receive support from relatives to perform daily activities, even though this possibility is low for those who are a widow, have no children or siblings. 

Regarding SN satisfaction, the SHARE results highlight that it is high for about half of respondents, even though daily contacts are not very frequent. The literature [30] indicates that decisions regarding housing in later life are also dependent on the quality of social relationships. Other authors [86] found in turn that older residents’ satisfaction in this respect depends on the network size and tie direction. Overall, our mixed results could relate to the fact that residents without some cognitive impairment can keep satisfactory connections with their own SN despite the access in a nursing home, whereas residents with a cognitive impairment do not. Similarly, Lapane et al. [87] highlight that seniors in an institution with a moderate/severe cognitive impairment had reduced odds of social connectedness than those with a mild/intact cognitive function. Moreover, according to Gaugler et al. [88], the fact that residents can receive help/assistance from relatives in residential settings (e.g., for both personal and instrumental activities) could provide a potential positive perception/satisfaction in seniors about their own SN.

### 4.3. Health and Functional Status

SHARE data highlight that health is perceived, above all, to be fair–poor, with an overall good–fair memory, even though long-term illnesses, chronic health conditions (e.g., Alzheimer’s), several drugs being taken and troubles with pain are reported by seniors. Some authors found that a low self-rated health is linked to nursing home admission [78,79], and, moreover, report that, in some cases, nursing home residents also tend to report having a good/very good health, even though generally they more frequently suffer from several diseases and limitations [80]. Cho et al. [89] found mixed results, with some older residents reporting satisfaction with improvements in their health (e.g., due to regular physical exercise), while others declared a worsened health (e.g., due to increasing weakness), and consequently, few hopes regarding their future health. A great part of the literature, however [61,78,79], confirms that seniors with overall poor health, functional limitations/cognitive impairment and a high number of drugs taken daily more frequently move into a nursing home in Europe. In particular, it can be difficult to manage older people with Alzheimer’s and Parkinson diseases at home, [41,90], and also stroke and osteoporosis can lead to accessing a nursing home, especially when seniors lose their autonomy due to these pathologies [61]. Bachmann et al. [81] add that often, a hospital discharge, after fall-related health problems, leads older people to enter into an LTC institution. Muszalik et al. [21], by comparing the clinical profile of frail seniors ageing in place and in nursing homes, also found, mainly in the latter setting, a higher number of diseases, higher duration of illnesses (over 15 years), higher use of a hearing aid, slightly higher use of glasses and higher number of medicines (six or more in a day). Overall, older people living in nursing homes often have multimorbidity and need poly-pharmacotherapy [91]. In particular, multimorbidity, as defined by the co-presence of two or more chronic conditions (e.g., heart failure/disease, stroke, diabetes, cancer, dementia, depression), is indeed prevalent in nursing home residents, and often it is, in turn, co-occurring with frailty, disability and polypharmacy. Moreover, older residents with multimorbidity and with hyper-polypharmacy have, respectively, a 35% and a 29% increased risk of mortality when compared to those not experiencing these conditions [92]. These older people need appropriate healthcare services, and nursing homes, however, representing a possible care solution in their daily living [93,94]. In particular, older people may need particular support for multiple drugs management and dosing at the right time, especially when their functional and cognitive capabilities are low, since drugs are difficult to be taken by seniors at home, with a great possibility of errors [95], especially when they live alone, with an older partner and/or without family and SN support. 

Data from the RePoSi register [96] also highlight the hard impact of diabetes and related comorbidities in older inpatients with multiple chronic conditions, who are hospitalised in internal medicine and geriatric wards. These seniors have a significantly high CIRS and take several drugs both at hospital admission, hospital discharge and at 1-year follow-up, with heart rate representing the crucial predictor of mortality during in-hospital admission. These findings support the issue of the critical impact of comorbidities, frailty and polypharmacy on the healthcare system, thus calling for a more personalised assessment and governance of older people at higher risk, in a continuum from hospital admission/discharge to post-discharge tertiary care [96]. 

Regarding functional abilities, a great part of older residents mapped by SHARE in Europe are severely impaired because of health, with both ADL/IADL and specific mobility limitations. Moreover, they frequently use aids (e.g., Zimmer frame or walker), and the fear of falling down and fatigue emerge as main aspects linked to their frail condition. Stolz et al. [50] report that seniors with severe functional limitations, and requiring long-lasting and intensive LTC, present a higher chance of accessing a nursing home in the last year of life. Some authors [61] stress in particular that ADL and IADL limitations are direct triggers for accessing a nursing home. Other authors [74] specify that 53% of residents in nursing homes have functional limitations and also a moderate cognitive impairment. They also report that several residents also experienced episodes of falls (36%) and urinary/bowel incontinence (42% and 265) within six months from admission in such arrangements.

The management of older people with multimorbidity and taking multiple medications, the identification of disease clusters and the evaluation of drug prescription appropriateness, via the means of a multidisciplinary team (internists and geriatricians, with primary care physicians), also involving patients themselves and their caregivers, represents a great challenge for a healthcare system aimed at protecting and supporting older people with LTC needs [96,97]. Overall frailty screening/detection and management is thus necessary via the means of personalised and evidence-based interventions aimed at identifying seniors needing additional support services, and at maintaining their physical and cognitive capacities; all this supported by feasible and cost-effective/efficient care policies and practices [98].

### 4.4. QoL and Other Psychological Issues

QoL is a wide concept including the persons’ physical/psychological health, social relationships and personal beliefs. It is defined by the WHO as “individuals’ perceptions of their position in life in the context of the culture and value systems in which they live and in relation to their goals, expectations, standards and concerns” [99] (p. 11). The majority of SHARE respondents overall report a low QoL and a moderate–low satisfaction with life. However, more detailed answers in this respect depict different emotions/feelings. On the one side, seniors indeed report they sometimes/rarely do the things they want to do, feel full of opportunities and think that the future looks good. Thus, they also felt sad/depressed last month. On the other side, they hardly ever/never felt isolated from others and lonely, and mention some hopes for the future. 

According to some of the literature, generally, institutionalisation represents a critical life event for older people, leading to a decreased QoL [81], especially in the case of frail seniors presenting with major depressive symptoms and a generally lower QoL [21]. Also, according to other studies [100], seniors living in nursing homes report lower self-esteem, higher depression and higher anxiety when compared with those ageing in place in their own homes. Further studies found that the QoL and self-esteem of older people living at home is higher than those living in institutions [101,102], and that seniors in nursing homes show overall modest levels of life satisfaction and happiness [61]. Some studies [103] also evaluated the relationship between multimorbidity and the health-related QoL (HRQOL) of adult patients in primary care, taking into account their medical conditions based on the CIRS. The findings revealed a robust link between increasing multimorbidity, especially when physical health is worsening, and a decreasing HRQOL. Other authors [104] highlighted how multimorbidity indexes predict a low HRQOL in older women, in addition to other aspects such as mortality and health services needed/used. Further authors [105] stressed in particular that several hospitalised patients in internal medical care wards reported a low self-rated health, thus indicating their need for support at admission, during the hospital stay and at discharge.

In contrast to this overall negative picture, other authors reported both positive and negative perceptions of older residents in nursing homes [89] impacting their perceived QoL. They found in particular that, among seniors’ perceptions of their daily lives in nursing homes, a reduction in loneliness emerged, since several participants reported their psychological comfort as improved since their admission to such care arrangements, also with “meaningful interpersonal relationships” [89] (p. 498) with nurses and other older residents, especially for those living alone before being institutionalised. Moreover, some residents “enjoyed being freed from their familial responsibilities, as they no longer needed to worry about meals and housekeeping while staying in the nursing home” [89] (p. 498). However, “They also worried about becoming a burden to their children and therefore did not inform them of their wish to go home” [89] (p. 500). However, autonomy remains a crucial aspect positively linked to older residents’ perceived QoL in nursing homes [106]. The further literature reports that individual relationships with residents and nursing staff could reduce the loneliness of seniors, in addition to providing “a sense of belonging to a community compared to spending time alone at home” [107] (p. 7).

### 4.5. Limitations and Strengths

Our study has some limitations to be considered. First of all, we present only a simple descriptive analysis, which could provide more insights via the means of further statistical analysis. Also, only data at an overall European level were analysed, without comparing single countries. Secondly, we did not provide a comparison regarding gender, and especially corresponding data on the same metrics on widowed women aged 80 and over ageing in place are lacking. However, this was decided since many missing data (refusal/do not know) were found, and thus, in the light of a possible/future more in-depth analysis in this respect, a preliminary picture of older Europeans living in nursing homes was considered as exploratory for further cross-country and cross-sectional examinations. In particular, this preliminary and detailed descriptive study can be used as a starting point for selecting interesting dimensions to be included in a possible multidimensional model for causal analysis. However, as supported by the literature [108], descriptive analysis can be considered as “stand-alone” research, with an important contribution to knowledge and practice, by providing simple data which can help readers to understand a phenomenon. Also, the cross-sectional nature of this study precluded the establishment of a causal relationship between dimensions that conversely, is allowed by longitudinal studies.

Regarding the SHARE survey, even though this paper considers data from Wave 8/2020 that was carried out in 27 European countries (and Israel), the representativeness of the overall respondents was compromised by the considerable effects of mortality due to the COVID-19 pandemic in the first six months of 2020, especially in nursing homes, and the consequent lack of refreshment samples in this regard. In addition, “Differences in sampling frames used across countries, can lead to country-specific under-coverage of the nursing home population” [52] (p. 25). Also, the SHARE data do not allow for differentiating between public and privately funded LTC nursing homes [41], and this limitation, combined with a lack of comprehensive description of LTC funding, structure and intent in differing parts of Europe, make our results challenging to interpret and ultimately apply. It is worth further consideration that the low response rates in this SHARE survey (Wave 8/2020) in turn limit the generalisability of the findings, especially with regard to those on public health and social policy issues [53].

The use of the SHARE survey has, however, some strengths to be considered. As anticipated above in the Methods section, contrary to several many other similar studies, SHARE includes persons living in nursing homes and residential care facilities. Also, respondents are followed when accessing such institutions and recorded as such [52]. In particular, persons aged 50 years or older, who are living in these facilities, are included in the SHARE target population. SHARE has also developed “special targeted measures to help interviewers gain access to nursing home respondents” [52] (p. 25). Nursing homes are indeed settings that are almost difficult to explore in some countries. Overall, a further strength of SHARE is the broad/extensive range of information from various contexts and cultures, thus making the data “extremely valuable and a stand-alone example in the world of social science surveys” [53] (p. 999]. Finally, although the COVID-19 pandemic had a negative impact on the coverage of the Wave 8 survey, and further reflection on the overall repercussion of this health emergency on the robusticity of the data could be provided, the SHARE survey made it possible to have information on a fragile segment of the population even in such a critical period. 

## 5. Conclusions

Our results provide a detailed socio-demographic, functional and psychosocial snapshot of older European residents in nursing homes, as captured by the SHARE survey, Wave 8/2020. They are mainly aged 80 years and over, female, widowed, living in Central/Northern Europe, with a low education level and a small SN size. Moreover, health is perceived, above all, as being fair–poor, several chronic illnesses and functional limitations are reported, in addition to an overall low QoL in many cases. A great part of the literature indicates that older people prefer ageing in place, thus maintaining independence and a better QoL [82], and trends at the EU level (especially in Nordic countries) show a process of de-institutionalisation put in place with this aim. Despite this, ageing in place could not be the definitive or better solution for older people with LTC needs, since possible life events in later life could impact its suitability. Residential care thus represents a possible replacement solution to family care, with potential benefits for both the cared for and caregivers [109]. It is, however, important to adopt an approach considering the results mentioned above by providing adequate and integrated formal/informal care arrangements, with residents keeping contacts with their relatives [100]. Informal care is not indeed fully replaced by entering, even permanently, a nursing home [80]. Social and health services should also collaborate to avoid premature and unnecessary institutionalisations [81]. Thus, it seems crucial that researchers, policymakers and managers of nursing homes join forces/cooperate in order to find a better allocation for the limited LTC resources [74], since nursing home admission is often linked to high public spending [81]. Public health programmes should also pay particular attention to depressive symptoms in older residents and support their mental health and overall wellbeing [100].

Despite limitations, our findings could be of help to train health professionals and all those working with frail older people, but call for further surveys on nursing home access and stay, thus potentially also driving the research towards the exploration of new housing solutions for seniors (e.g., co-living arrangements). Also, more research on informal care provision in nursing homes is welcome. Overall, more future research is needed in order to carry out dedicated surveys on older residents in nursing homes, aimed at providing findings “inspiring” government agendas for better supporting these frail seniors in order to improve their living in such care facilities in Europe. This would contribute to the effective implementation of European initiatives to strengthen caring and LTC systems, e.g., the European Care Strategy (ECS) and the European Commission–World Health Organization (EC-WHO) partnership on LTC, launched to support people needing LTC, as well as their caregivers, and to improve access to affordable high-quality and resilient LTC in the EU Member States [110,111].

## Figures and Tables

**Table 1:** Topics and variables used in this study.

Topics	Variables
Length of stay in nursing homes	Years in accommodation
Socio-demographic characteristics	Country
	Age group
	Gender
	Marital status
	Household size
	Educational level
	Income sources
	Income used to cover nursing home cost
Family members	How many brothers alive
	How many sisters alive
	How many children
	How many grandchildren
Overall social network (SN)	SN size
	SN satisfaction
	Type/number of persons in the SN
	Gender of persons in the SN
	SN contacts
Health	Self-perceived health
	Memory
	Presence of long-term illness
	Number of chronic health conditions
	Type of diseases suffered
	Taking at least five different drugs in a typical day
	Wears glasses/contact lenses
	Troubled with pain
Functional status	Limited in activities because of health
	Number of ADL limitations ^1^
	Number of IADL limitations ^2^
	Number of mobility limitations
	Use of aids
	Aspects affected by frailty
Quality/satisfaction of/with life	Quality of life (QoL) and wellbeing
and other psychological aspects	Satisfaction with life
	”Do the things you want to do” ^3^: sometimes/rarely
	Feel full of opportunities: sometimes/rarely
	”Future looks good” ^3^: sometimes/rarely
	Feel full of energy: rarely/never
	”Age prevents from doing things” ^3^: often/sometimes
	Sad or depressed last month: yes
	Feel isolated from others: hardly ever or never
	Feel lonely: hardly ever or never
	Some hopes for the future mentioned: yes
	Suicidal feelings or wish to be dead: no

^1^ Activities of daily living (ADL); ^2^ instrumental activities of daily living (IADL); ^3^ these are the original questions included in the Survey of Health, Ageing and Retirement in Europe (SHARE).

**Table 2:** Length of stay in nursing home (absolute values/n and %).

Length of Stay ^1^	n = 485	%
Years in accommodation:		
1	128	26.4
2–3	100	20.6
4–6	69	14.2
7–11	55	11.3
12–20	26	5.4
21–42	15	3.1
Missing (refusal/do not know)	92	19.0

^1^ Only the higher values are reported (first to third).

**Table 3:** Socio-demographic characteristics (absolute values/n and %).

Characteristics ^1^	n = 485	%
Central and Northern Europe ^2^	349	72.0
Age group: ≥80 years	356	73.4
Gender: female	330	68.0
Marital status:		
Widowed	269	55.5
Married	120	24.7
Household size: 1 member	405	83.5
Education (n. years attending full-time education):		
6–8	136	28.0
9–13	190	39.2
Education ISCED 2011 ^3^:		
Primary/less	184	37.9
Secondary	214	44.1
Post-secondary	87	17.9
Income sources ^4^:		
Public old-age pension	407	83.9
Public survivor pension from spouse/partner	112	23.1
Income used to cover nursing home cost ^4,5^:		
Pensions	311	64.1
Savings	61	12.6
Public long-term care (LTC) service	43	8.9

^1^ For each dimension, only the higher values are reported (first or first to second, depending on the topic), apart from ISCED 2011; ^2^ Austria, Germany, Sweden, the Netherlands, France, Denmark, Switzerland, Belgium, Luxembourg, Finland, Czech Republic; ^3^ educational level is measured by the International Standard Classification of Education (ISCED-2011); ^4^ multiple choices were possible; ^5^ missing = 150.

**Table 4:** Family members (absolute values/n and %).

Aspects ^1^	n = 485	%
How many brothers alive ^2^:		
Nobody	111	22.9
One	107	22.1
How many sisters alive ^3^:		
Nobody	106	21.9
One	119	24.5
How many children:		
Nobody	90	18.5
One	110	22.7
Two	144	29.7
How many grandchildren:		
Nobody	110	22.7
Two–three	115	23.7
Four–five	108	22.3

^1^ For each dimension, only the higher values are reported (first to second or first to third, depending on the topic); ^2^ missing = 197; ^3^ missing = 170.

**Table 5:** Social network (SN) (absolute values/n and %).

Aspects ^1^	n = 485	%
SN size		
Network less ^2^	99	20.4
Small SN (1–2 members)	243	50.1
Large SN (3+ members)	143	29.5
SN satisfaction (range 0–10) ^3^:		
High (9–10)	236	48.7
Moderate (7–8)	112	23.1
Type/number of persons in the SN:		
Children (1–2 members)	221	45.6
Sibling (1–2 members)	49	10.1
Friends (1–2 members)	78	16.1
Gender of persons in the SN:		
Women (1–2 members)	261	53.8
Men (1–2 members)	205	42.3
SN contacts (closest members, e.g., children):		
Daily contact	182	37.5
Several times/weeks	107	22.1
1 time per week	65	13.4

^1^ For each dimension, only the higher values are reported (first to second or first to third, depending on the topic), apart from SN size and gender of persons in the SN (with, however, 19 missing in the latter dimension); ^2^ network less/0 = no persons in the SN; ^3^ “0” = completely dissatisfied; “10” = completely satisfied.

**Table 6:** Health (absolute values/n and %).

Aspects ^1^	n = 485	%
Self-perceived health ^2^:		
Good	110	22.7
Fair	179	36.9
Poor	153	31.5
Memory ^2^:		
Good	136	28.0
Fair	134	27.6
Poor	93	19.2
Presence of long-term illness	374	77.1
Number of chronic health conditions ^3^		
1	114	23.5
2	108	22.3
3–6	199	41.0
Type of diseases suffered ^4^:		
High blood pressure or hypertension	190	39.2
Osteoarthritis	125	25.8
Alzheimer’s disease, dementia	123	25.4
Taking at least five different drugs in a typical day	243	50.1
Wears glasses/contact lenses	367	75.7
Troubled with pain	239	49.3

^1^ For each dimension, only the higher values are reported (first, first to second, first to third, depending on the topic); ^2^ these dimensions are categorised as excellent, very good, good, fair, poor; ^3^ this definition contains both physical and mental health conditions; ^4^ multiple choices were possible.

**Table 7:** Functional status, ADL, IADL and use of aids (absolute values/n and %).

Aspects ^1^	n = 485	%
Limited in activities because of health:		
Severely limited	276	56.9
Limited, but not severely	139	28.7
Number of ADL limitations ^2^ (range 0–6):		
0	169	34.8
1–2	118	24.3
3–5	113	23.3
6+	85	17.5
Number of IADL limitations ^3^ (range 0–7):		
0	95	19.6
1–2	49	10.1
3–5	87	17.9
6+	254	52.4
Number of mobility limitations ^4^ (range 0–10):		
1–2	61	12.6
3–4	61	12.6
5–8	195	40.2
Use of aids ^5^:		
Zimmer frame or walker	194	40.0
Incontinence pads	194	40.0
Bars/grabs/rails	170	35.0
Aspects affected by frailty ^5^:		
Dizziness, faints or blackouts	185	38.1
Fear of falling down	183	37.7
Fatigue	178	36.7

^1^ For each dimension, only the higher values are reported (first to second or first to third, depending on the topic), apart from ADL and IADL; ^2^ ADL, basic activities of daily living; ^3^ IADL, instrumental activities of daily living; ^4^ mobility limitations regarding arm function and motor; ^5^ multiple choices were possible.

**Table 8:** Quality of/satisfaction with life and other psychological aspects (absolute values/n and %).

Aspects ^1^	n = 485	%
Quality of life (QoL) and wellbeing (CASP-12 index) ^2^:		
Low < 35	173	35.7
High ≥ 35	138	28.5
Satisfaction with life (range 0–10) ^3^:		
Moderate (7–8)	143	29.5
Low (0–6)	129	26.6
Other psychological aspects ^4^:		
“Do the things you want to do”: sometimes/rarely	215	44.3
Feel full of opportunities: sometimes/rarely	218	44.9
“Future looks good”: sometimes/rarely	198	40.8
Feel full of energy: rarely/never	213	43.9
“Age prevents from doing things”: often/sometimes	289	59.6
Sad or depressed last month: yes	195	40.2
Feel isolated from others: hardly ever or never	260	53.6
Feel lonely: hardly ever or never	199	41.0
Some hopes for the future mentioned: yes	232	47.8
Suicidal feelings or wish to be dead: no	293	60.4

^1^ For each dimension, only the higher values are reported (first or first to second, depending on the topic), apart from Control, Autonomy, Self-realization, and Pleasure (CASP-12) index; ^2^ CASP-12 = QoL Index, with higher scores indicating better QoL. Missing = 174; ^3^ “0” = completely dissatisfied; “10” = completely satisfied; ^4^ for these dimensions, possible responses were often/sometime/rarely/hardly ever or never, in addition to yes/no and do not know. In particular, the frequency of do not know was rather high, with “n” ranging 95–127.

## Data Availability

This paper uses data from SHARE Wave 8. Release version: 8.0.0. SHARE-ERIC. Dataset. DOI: 10.6103/SHARE.w8.800. For more detail on SHARE see Börsch-Supan et al., 2013. For methodological aspects, see Bergmann and Börsch-Supan (Eds.), 2021. Further information on eligibility for the study can be found in the SHARE Release Guide that is publicly available on the SHARE website (www.share-project.org (accessed on 23 January 2023). Data access is publicly granted upon completion of the application form: http://www.share-project.org/data-access/user-registration.html (accessed on 23 January 2023).
